# “Case Report: Klinefelter Syndrome Presenting With Dystonic Tremor: Expanding the Phenotypic Spectrum and Therapeutic Option”

**DOI:** 10.5334/tohm.1084

**Published:** 2025-12-05

**Authors:** Debayan Dutta, Jacky Ganguly, Purba Basu, Soumava Mukherjee, Hrishikesh Kumar

**Affiliations:** 1 Movement Disorder Centre, Department of Neurology, Institute of Neurosciences, Kolkata, India

**Keywords:** dystonia, dystonic tremor, Klinefelter Syndrome, Chromosomal aneuploidy, Botulinum toxin

## Abstract

**Background:**

Klinefelter syndrome (KS) is associated with tremor and dystonia, but the exact phenomenology and neurophysiology are not well described.

**Case Report:**

We present a case of a 51-year-old married male who presented with segmental dystonia affecting the bilateral upper limbs and lower face, accompanied by perioral dyskinesia and a dystonic tremor that was more pronounced on the left side. There was no family history of similar symptoms. His evaluation revealed a history of infertility and the presence of gynecomastia, prompting further endocrine assessment, which demonstrated hypergonadotropic hypogonadism. This finding guided the diagnosis of Klinefelter syndrome (KS), confirmed by a 48,XXY karyotype. He was treated with antitremor medications and Botulinum toxin (BoNT-A), which led to moderate improvement.

**Discussion:**

Our case represents the fourth reported case of KS with dystonic tremor in the literature.

## Case report

A 55-year-old male presented to our movement disorders clinic with posturing of the left upper limb for the last year, followed by the right upper limb over the past three months. This was associated with mild tremulousness of the left upper limb while reaching for objects. He also noticed involuntary abnormal movements of his lips and face during the same period. There was no diurnal variation, sleep-related improvement, or paroxysmal exacerbation of symptoms. He had no family history of similar illness and no history of chronic intake of alcohol, antibiotics, or neuroleptic agents.

There was a history of infertility, and gynecomastia was noted on general examination. Cognitive assessment showed a score of 28/30 on the Mini-Mental State Examination (MMSE). On examination (Video 1), he exhibited bilateral upper limb mobile dystonic posturing of the index finger, evident both while seated and during ambulation. This resembled the *pointing index phenomenon*, a form of subtle focal hand dystonia [1]. The abnormal movements could be mitigated by restraining his left ring finger with a rubber band or by placing heavy weights in his left hand (sensory tricks or geste antagoniste). He also displayed an increased blink rate and involuntary tongue protrusion with pouting movements of the lips, suggestive of orofacial dyskinesia. Tremor of the upper limbs was absent at rest but most prominent with the arms outstretched, and occurred in conjunction with dystonic posturing of the affected part, suggestive of dystonic tremor. Cerebellar examination showed impaired tandem gait, while the rest of the neurological examination was normal.

**Video 1:** Video shows bilateral distal upper limb dystonic posturing (left more than right) with orofacial choreiform dyskinesia with upper limb course dystonic tremor on outstretched hand. The patient used to wrap a rubber band around his left ring finger or keep some weight like his mobile phone in his left hand to alleviate the abnormal posturing (Geste anagoniste/sensory trick). Movement used to aggravate while typing. His gait was normal except impaired tandem gait.

Routine hemogram and biochemical investigations were unremarkable. Endocrine evaluation demonstrated low serum testosterone with elevated LH and FSH, while other hormonal assays, including serum TSH and cortisol, were normal. MRI brain showed mild cerebellar atrophy. The comprehensive SCA panel for trinucleotide CAG repeats (SCA 1, 2, 3, 6, 12) was negative. Whole-exome sequencing revealed no pathogenic variants. In view of the combination of tremor, dystonia, hypergonadotropic hypogonadism, and gynecomastia, karyotyping was persuaded revealing the presence of an additional X chromosome (48, XXY).

He was treated with propranolol (40 mg/day) and trihexyphenidyl (6 mg/day). Botulinum toxin (BoNT-A) was injected into the left 2nd, 3rd, and 4th lumbricals (5 units each), resulting in moderate improvement in the left hand dystonia.

## Result

We conducted a literature search in the PubMed and MEDLINE databases using the following terms: “klinefelter syndrome”[MeSH Terms] OR “Klinefelter”[All Fields] AND “syndrome”[All Fields]) AND “tremor” OR “tremors” “tremorous”[All Fields] OR (“dystonia”[MeSH Terms] OR “dystonia”[All Fields] OR “dystonic disorders”. A total of 51 patients were identified across 18 articles. Video recordings were available for 5 patients, which were analysed. Five articles included at least one movement disorder specialist among the authors, ensuring expert assessment of tremor phenomenology. Electrophysiological evaluation was performed in two of them [2,3].

Among 22 cases with documented onset age, the mean age at onset was 12.1 years. The most common karyotype was 47, XXY (33/50, 66%), followed by 48, XXYY (13/50, 26%). Other karyotypes included mosaic 47, XXY/48 XXXY (1/50, 2%), mosaic 46, XY/47, XXY (1/50, 2%), and 49, XXXXY (1/50, 2%). A family history of tremor was present in 12 patients (23.5%). Alcohol responsiveness was documented in one case.

### Tremor characteristics

Upper limb tremors were the most frequent finding, followed by head tremor (6/51, 11.7%) and voice tremor (1/51, 1.9%). The most common pattern was a combination of postural and kinetic/intention tremor (15/51, 29.1%). Isolated postural tremor occurred in 14 patients (27.1%), and dystonic tremor was noted in 3 patients. Rest tremor was seen in 10 patients (19.6%). Two had a typical rest tremor of 5–6 Hz, and one had a lower-frequency (3–5 Hz) rest tremor. All were associated with either postural or kinetic tremor. No correlation was found between tremor type and chromosomal aneuploidy.

### Other Neurological features

Subnormal intelligence was reported in 9 patients, although formal IQ scores were not available. Ataxia and dysarthria were each reported in 5 patients (9.8%). Epilepsy and nystagmus were seen in 2 patients (3.9%), while migraine was reported in 1 (1.9%). Psychiatric symptoms, including ADHD(Attention Deficit Hyperactivity Disorder) and bipolar disorder, were reported in three patients.

### Systemic features

Gynecomastia was the most frequently observed systemic feature (13/51, 25.4%). Additional findings included testicular atrophy (7, 13.7%), varicocele (1, 1.9%), infertility (1, 1.9%), hypogonadism (7, 13.7%), cryptorchidism (1, 1.9%), mitral valve prolapse (1, 1.9%), cystic fibrosis (1, 1.9%), and skeletal abnormalities such as kyphoscoliosis in 3 patients.

### Treatment response

Tremor associated with KS was managed similarly to essential tremor (ET). Standard anti-tremor medications effective in ET were tried in most cases; however, the therapeutic response was variable. First-line therapy, including non-selective beta blockers like propranolol was tried in 7 patients. Among them 4(57.1%) did not improve, 2(28.5%) had mild improvement and 1(14.2%) had significant improvement. Primidone was tried in 8 patients, and only one patient had a moderate response, and it was ineffective in the rest 7 patients.

Testosterone replacement therapy (TRT) was administered to 16 patients with documented testosterone deficiency. Improvement in tremor was seen in 7 patients (47%), while 5 (33%) experienced no benefit. Deep brain stimulation (DBS) targeting the ventral intermediate nucleus (VIM) was performed in 4 reported cases. Our patient represents the first documented case of KS treated with Botulinum toxin (BoNT-A) and had a moderate response to dystonic tremor.

## Discussion

We have described a patient with dystonic and postural tremor associated with KS, successfully treated with BoNT-A.

KS is a chromosomal aneuploidy characterized by the presence of an additional X chromosome in males with features of hypogonadism like infertility and gynecomastia [4], It was first described by Dr Harry Klinefelter [4], KS can occasionally present with two or more extra X chromosomes, referred to as poly-X variants. In a study by Harlow et al, it was shown that 63.4% of KS patients have tremors compared to 13.7% of the general population [5]. The tremor phenomenology is heterogeneous – often resembling essential tremor, though dystonic features can also be present. Tremor is not unique to KS but is also reported acrossother aneuploidy syndromes (47XYY, 48XXYY, 49XXXXY, mosaic XXY/XXXY) [6].

Many of the published reports are from earlier decades, when video documentation was not available. As a result, reanalysis of those cases was not possible, and the limited clinical descriptions often prevented precise characterization of the tremor phenomenology. Among cases with reliable documentation, the mean age of onset of tremor was in the second decade. All patients had upper limb tremor with/or head tremor, voice tremor, and lower limb tremor [6]. The majority of patients had a symmetrical, distal upper limb predominant postural/action tremor with intention component in the absence of rest tremor, which clinically and electrophysiologically resembles ET [7,8]. However, a subset of patients had a course rest and postural tremor with dystonic posturing of the affected or unaffected body part with a negative weight load test, which can be classified as tremor with dystonia [9,10,11].

Ataxia was present in 10% of patients with the 48, XXYY karyotype [12]. Although the X chromosome carries the *FMR1* gene – implicated in Fragile X–associated tremor/ataxia syndrome (FXTAS) – ataxia in KS does not appear to be related to *FMR1* repeat expansions. This is because the additional X chromosome in KS is typically inactivated in KS [13] and at least one case report specifically tested for the *FMR* premutation, which was negative [7]. Epilepsy was documented in two patients, with the age of onset of epilepsy preceding that of tremor by 1–2 decades [14,12]. Myoclonus was also noted in one patient with mild ataxia, which improved significantly after testosterone replacement [15]. Other neuropsychiatric symptoms, like ADHD, was noted in two patients; both had a very early age of onset of tremor and followed by the onset of ADHD within the 2^nd^ decade. Their tremor was medically refractory, and both underwent VIM DBS, resulting in favourable outcomes [16,3].

Preliminary evidence from structural neuroimaging in KS has shown reduced brain volume in frontal, temporal lobes, and cerebellum [17]. However, two case reports mention T2/FLAIR hyperintense lesions in frontotemporal periventricular white matter, subcortical region with extension into the ipsilateral basal ganglia [18,19]. Cerebellar volume reduction appears to be a consistent finding and is thought to contribute to the ET-like tremor seen in KS, likely reflecting a gene-dosage effect [20]. Functional neuroimaging often shows evidence of reduced BOLD (blood-oxygen-level-dependent) activity in functional MRI in the left temporo-parietal region [17].

The first-line drugs were beta blockers, which showed mild to moderate improvement [3,21]. Primidone was less effective [18,16,8,14]. Other medications like benzodiazepine, topiramate were variably effective [14]. Treatment with testosterone replacement therapy yielded variable results, with improvement [22,8], worsening [23] and no effect [7,3,24,25], all being reported.

Our case may be the first in the literature where dystonic tremor in a patient of KS was managed with Botulinum toxin A.

VIM nucleus of the thalamus was chosen as DBS target in 4 cases, bilaterally in 3 patients and left thalamic DBS in one patient due to asymmetric right upper limb tremor. Stimulation parameters were documented in one case in which lower contacts (contact 1a,1b,1c on the right side and contact 8 on the left side) were used with a high frequency (145 Hz) stimulation. Post-operative complications like recurrent skin ulceration around the pacemaker was documented. All of the patients had sustained improvement in tremor and quality of life with immediate effect [16,14,3,25].

Among other aneuploidy syndromes, Jacob’s syndrome (JS)(47, XYY) was associated with mild rest and/or intention tremor in 43% (39/90) patients. These tremors were typically non-disabling and occasionally interfered with handwriting [26]. However, in a case reported by Davis et al, one patient with JS had significant upper limb tremor with cervical dystonia, writing dystonia and ataxia [27]. Compared to classical KS, patients with JS have higher tremor amplitude and greater variability in tremor frequency [2].

Another KS variant, 48, XXYY syndrome patients can present with tremor, ataxia, ADHD, cardiac and dental abnormalities, and radioulnar synostosis. In the largest cohort (n=95) of 48, XXYY patients, intention tremor was present in 60% patients [26]. Tremor is usually mild beginning around puberty [12,28].

Mosaicism was also described to have an association with tremor. One young 18-year-old patient with a mosaic chromosomal pattern of XXY/XXXY presented with bilateral postural tremor with intellectual disability, and dysmorphic features including high arched palate and short 5^th^ finger [29].

The aetiology of tremor in KS and other aneuploidy syndromes has been the subject of several hypotheses. Testosterone and other androgens are required for lower motor neuron growth and development [30]. Hence, their deficiency was initially proposed as a key mechanism underlying tremor in KS. However, the occurrence of tremor in patients with normal testosterone levels, along with the inconsistent therapeutic benefit of testosterone replacement, argues against a purely androgen-deficiency – based explanation.Several genes located on the X chromosome have been implicated in tremor disorders – including *FMR1* in FXTAS, the androgen receptor gene in Kennedy’s disease, and *PLP1* in the Xq22–27 region associated with Pelizaeus–Merzbacher disease – raising the possibility that X-linked genetic influences contribute to the phenotype. Another hypothesis brought forward was the escape of X-chromosomal inactivation or the overexpression of Y-chromosomal genes, resulting in a gene-dosage effect [31].

The strength of our study lies in the comprehensive search strategy focused on a specific neurological manifestation of KS, enabling us to compile a relatively large cohort of 51 reported cases. However, much of the available information is derived from isolated case reports, leading to substantial heterogeneity and a high likelihood of publication bias. Consequently, the strength of the conclusions that can be drawn remains limited. Long-term follow-up data were unavailable, and detailed family information was insufficient to explore intrafamilial phenotypic variability. Moving forward, we aim to establish a dedicated database to systematically analyse the neurophysiological mechanisms underlying tremor in KS, with the goal of improving both medical and neuromodulation-based therapeutic strategies.

## Conclusion

Even in the current era of advanced genetic testing, structural chromosomal disorders such as Klinefelter syndrome should not be overlooked. In a male patient with early onset tremor, ataxia with or without dystonia, aneuploidy syndromes must be considered – particularly when accompanied by red flags such as a positive family history, intellectual disability, infertility, gynaecomastia and testicular atrophy. Management includes standard anti-tremor medications, testosterone replacement therapy, Botulinum toxin for dystonic tremor, and DBS, all of which have demonstrated variable but encouraging results. Further studies are needed to clarify the underlying neurophysiological mechanisms and to guide more targeted therapeutic strategies.

## Data Accessibility Statement

Patient-related data can be made available on request from the authors. Other theoretical data is available in the open public domain that includes a DOI.
